# Impact of COVID-19 pandemic on the mental health of school-going adolescents: insights from Dhaka city, Bangladesh

**DOI:** 10.1016/j.heliyon.2022.e09223

**Published:** 2022-03-29

**Authors:** Ridwan Islam Sifat, Maisaa Mehzabin Ruponty, Md. Kawser Rahim Shuvo, Mehjabin Chowdhury, Shidratul Moontaha Suha

**Affiliations:** a Department of General Education, Faculty of Humanities and Social Science, Northern University Bangladesh, Dhaka, Bangladesh; b Department of Development Studies, Faculty of Arts and Social Sciences, Bangladesh University of Professionals, Dhaka, Bangladesh; c Faculty of Social Sciences, Eötvös Loránd University (ELTE), Budapest, Hungary

**Keywords:** COVID-19, Adolescents, Mental health, Online class

## Abstract

The pandemic has affected every walk of life, and mental health is no exception. Bangladesh has been operating under a resource crisis, and this crisis has incurred and is incurring a governance priority dilemma. Unending vacations of the educational institutions are taxing our students' mental serenity, and among those, adolescents are more vulnerable. Unending leaves of the educational institutions are taxing our students' mental peace, and among those, adolescents are more susceptible. Across the globe, a good number of studies have been performed, and Bangladesh is no exception. However, adolescents have received less attention in those studies, and this paper fills the gap. This explorative study opted for a qualitative method that covered data collection like in-depth interviews among 60 respondents. This study aims to simultaneously unveil the causes of mental dissonance among adolescents and the impact of infection prevention measures (e.g., lockdown) on adolescents' mental health in the capital city of Bangladesh. This study also recommends a possible way out of this crisis. The study revealed that prolonged school closure, fear of the disease, disruption in education, excessive use of digital devices, and the culture of ignoring adolescents' mental health are responsible for pausing detrimental effects on adolescents' mental health. Adolescents suffered from mental health issues like stress, anxiety, depression, and sleeping disorders during the lockdown.

## 1 Introduction

The novel coronavirus originated in a city in China and spread throughout the world. Several countries declared the pandemic a national emergency, forcing millions of people to go into lockdown (Ahmed and Sifat, 2021a). Most countries have attempted to impose "social distancing" among people to reduce infection and the number of deaths. Many countries imposed lockdowns or even closed borders for some time. As part of the lockdown, many schools have been closed, and the school has adapted to online classes, online exams, etc., reducing the interactions with their peers and leading them to fewer chances of physical activity. These situations may have an impact on mental health and welfare along with mental issues like anxiety, stress, depression, and sleeping difficulties (Ahmed & Sifat, 2021a, 2021b; Saha et al., 2021; Sifat, 2020, 2021a).

A study done by the Center for Disease Control and Prevention found that anxiety has been diagnosed in around 4.4 million adolescents between the ages of 13 and 17 years. 1.9 million adolescents have been diagnosed with depression because of home quarantine due to COVID-19 (Galvin, 2020). From May 17, 2020, the government of Bangladesh has imposed a lockdown and declared all educational institutions and schools closed sine die. More than a million teachers and 3.7 million students are confined at home (Ahmed, 2020). The role of the school is essential; it provides educational lessons and resources to adolescents and creates the opportunity to communicate with their teachers, which acts as psychological help. Evidence shows that whenever adolescents are beyond schooling, physical activity decreases, they use digital devices more than usual, sleeping habits change, and they resort to an unhealthy diet, which increases weight and performance in cardio and respiration drops (Brazendale et al., 2017). Fear of infection, a lack of personal space at home, monotony, a lack of knowledge, a decrease or loss of family income, or a decrease or loss of family income can all cause problems for little minds. A study done on the effect of the COVID-19 pandemic on adolescents' mental wellbeing in Bangladesh is worrying. According to a survey of parents with primary-aged adolescents, 87% reported that their adolescents were missing school and less than half stated that they were feeling lonely, which altogether affects their adolescents’ mental health and wellbeing (Yeasmin et al., 2020).

Previous research has found that general practitioners are involved in all stages of the virus response, including helping to prevent viral transmission by tracking subjects. They are reducing the number of cases by treating patients, providing medical surveillance, and caring for patients' clinical and psychological well-being so that the situation can return to normal. Furthermore, anxiety-like reactions to the COVID-19 pandemic may shift to feelings of hopelessness and depression linked to adverse outcomes, including suicidal behavior (Serafini et al., 2020). Existing research indicates that people with major affective disorders might have long-term difficulties processing sensory information, which has been associated with higher levels of depression, impulsivity, alexithymia, and hopelessness (Serafini et al., 2016). The most prevalent psychiatric disorder is major depressive disorder, which contributes significantly to the global disease burden. The major effects of depression, such as deteriorating physical health and suicide, are caused by this burden (De Berardis et al., 2018).

### 1.1 Background and problem statement

The coronavirus causes diseases like the common cold and severe diseases like severe acute respiratory syndrome. Basic hygiene should be practiced, such as washing hands religiously with water and soap as recommended by the World Health Organization (World Health Organization, 2021). Hygiene should be maintained while sneezing or coughing. "Physical distancing" will be imposed by keeping at least 1 m (three feet) distance between each other. There were varying national responses to the COVID-19 pandemic, including containment measures such as lockdowns, quarantines, and curfews. As the novel coronavirus continues to spread, some countries impose different types of lockdowns on their residents. COVID-19 lockdown measures have partially or fully closed schools for more than 90% of the world's student population across 186 countries and territories, according to UNESCO (Wiley, 2020).

Although quarantine was mandatory to stop the spread of the disease, it affected the lives of adults and adolescents in many ways. In addition to the threats to public health and economic and social turmoil, it threatens the long-term life and wellbeing of millions of people. As of February, 28 countries have closed pre-primary, primary, lower-secondary, upper-secondary levels, and tertiary education worldwide due to the COVID-19 pandemic, which affected 221,964,329 learners (UNESCO, 2021). Many countries, therefore, have resorted to online training or home-based learning. These students are experiencing further distress due to inadequate help and attention from the trained instructors, making education more expensive for them and their families. They need to utilize additional time, support, and resources. Due to the closing of schools, students' interaction and communication with schoolmates, play, exercises, and peer activities are hindered, which have proven vital for the growth, development, and learning of young human minds. The ones at the most significant risk are the youngest, as their brains are still developing and exposed to high levels of stress and isolation, which can lead to permanent abnormal development. Adolescents exposed to stressors such as separation through isolation from their families and friends, seeing or being aware of critically ill members affected by coronavirus, the passing of loved ones, or even thinking of their death from the virus can develop anxiety, panic attacks, depression, and other mental illnesses (Saha et al., 2021; Sifat, 2021a).

With more than five lakh corona cases affected with hundreds of deaths daily even after 11 months after the first COVID-19 case in Bangladesh, schools in Bangladesh are less likely to open soon. Recently, the government of Bangladesh has decided to observe the months of February and will decide in March about the opening of schools (Alamgir, 2021). The government of Bangladesh enforced a full lockdown, and all schools were closed from May 17, 2020. This adversely affects the well-being of adolescents by interfering with their health care, diet, protection, schooling, and overall mental health (Bhuiyan et al., 2020). Yet, there is insufficient literature available in Bangladesh on the long-term impact of the COVID-19 pandemic on adolescents’ mental health.

The key objective of this analysis is to determine the effect of the COVID-19 pandemic on adolescents' mental health. Accordingly, it is essential to decide how prolonged school closure, social distancing, and the pandemic affect the psychological wellness status of adolescents. Hence, this study is intended to explore the associated factors that affect adolescents' mental health in Bangladesh. It will also attempt to provide some policy suggestions to address the mental health issues of adolescents in Bangladesh in general and during crises.

### 1.2 Research questions

1. What kind of impact is being created on the mental health of school-going adolescents due to the COVID-19 pandemic?
2. What are the factors impacting the mental health of school-going adolescents during the COVID-19 pandemic?
3. What policy suggestions can be offered to address the mental health issue of adolescents of Bangladesh in general and during crises situations in particular?

## 2 Literature review

### 2.1 Mental health of adolescents during COVID-19

According to a study of 1800 Chinese adolescents, one out of every five adolescents (20%) in China suffers from depression, anxiety, or both. Because of the COVID-19 pandemic, psychological well-being issues remain genuinely high among U.S. youngsters. Three-fourths of adolescents are suffering from depression and anxiety (Galvin, 2020). According to a survey of parents with primary-aged adolescents, 87% reported that their children missed school and that they felt alone, which impacted their adolescents' mental health and wellbeing (Rawstrone, 2020).

According to Yeasmin et al. (2020), many adolescents are suffering from mental health disturbances in Bangladesh during the lockdown period. Mothers' and fathers' ability to forestall their emotional pain or manifestation of depression from influencing their role as parents might be a significant source of resilience for their adolescents. Adolescents with more educated parents, adolescents living in cities, adolescents with both higher and lower family income, adolescents from smoking families, and adolescents with parental depressive symptoms are the study's most vulnerable groups (Mallik and Radwan, 2021).

### 2.2 Culture of ignoring mental health of adolescents

According to Aresfin and Shafiullah (2020), in Asian nations, the mental state has traditionally been given very little importance. Therefore, youngsters, thanks to the demographic cluster, are usually less vocal concerning their issues. Moreover, in line with the World Health Organization Assessment Instrument for Mental State Systems (WHO-AIMS) report from 2007, mental state expenditures from government health departments are insignificant and amount to 0 to 5 percent of the total outlay (World Health Organization, 2007). The government developed policies to deal with several problems long faced by youngsters. Unfortunately, the mental state was not one of those issues. As a standard thread, adolescents' mental state problems arising from COVID-19's onset have not received the attention they merit in South Asian countries (Yeasmin et al., 2020).

### 2.3 Inequality in access

A report by UNICEF (2020) found that almost a third of the world's school adolescents, 463 million kids globally, were unable to access remote learning once COVID-19 closed their faculties. This is in line with a brand-new United Nations agency report, as countries across the globe grapple with their "back to school" plans. At the peak of nationwide and native lockdowns, nearly 1.5 billion faculty adolescents were laid low by school closures. At least 120 million children are unable to be reached, owing to challenges and limitations in online learning for young children, a lack of remote learning programs for this educational class, and a lack of home assets for remote learning. The Remote Learning Reachability report identifies the limitations of distance learning and finds deep inequalities in inaccessibility.

### 2.4 Impact due to quarantine and separation from parents

In research done by Singh et al. (2020), it was found that COVID-19 infection is expressed differently in adolescents and adolescents. Yet, incidents of infection in minors have been reported worldwide, which result in adolescents being quarantined. There are many incidents where either the mother or father or both of them were infected and isolated. In either situation, children are separated from their parents. Many nations have imposed rigid lockdown policies as a means to fight the COVID-19 situation. For example, in China, a few grown-ups, teenagers, and kids have been placed in complete detachment to control the spread of disease. Although quarantining measures are for the benefit of the community at large, their psychological effects cannot be ignored. The kids in disconnection with parents need exceptional consideration as they may be in danger of creating psychological wellness issues because of the anguish brought about by the quarantine of parents (Liu et al., 2020). The role of parents is very important in the initial years of life of adolescents; any disturbance in the form of isolation from parents can have a long-lasting impact on the adolescents’ attachment. It has been discovered that detachment from the mother or father can make a kid more vulnerable and pose a danger to their emotional well-being (Dalton et al., 2020).

### 2.5 Economic hardship, food uncertainty and mental health

According to Bodrud-Doza et al. (2020), about 85.60% of adolescents are under COVID-19-related stress, which results in sleepiness, short temper, and chaos in the family. Fear of COVID-19, hampering study plans and future careers, and financial difficulties are the leading causes of human stress. The factors of economic hardship, human stress, and the food crisis are all connected altogether. It creates stress for many people. It also interferes with formal education and plans, causing stress for the job seeker.

An exploration of the current literature related to COVID-19 and the mental health of adolescents reveals that COVID-19, with the spread of the disease, also spreads fear in their minds too. Fear of the new disease, the economic problems of the parents, staying away from school and friends, loss of parents, pressure from online classes are creating a negative impact on adolescents' mental health in COVID-19. The conditions of anxiety, depression, and stress have worsened due to COVID-19. However, the result is mostly based on the interpretation of quantitative data. More qualitative data should include determining the severity of the issues and the depth of the problem. A mixed approach is needed to determine the issues most specifically.

## 3 Methodology

### 3.1 Research method

This research adopted qualitative research. However, there is a lack of rigorous study on this topic, especially in the context of Bangladesh. A qualitative method was chosen to explore the research question, as the study was aimed at exploring the impact of the COVID-19 pandemic on the mental health of school-going adolescents. Qualitative data has been used to understand the situation better and to know adolescents’ perceptions, experiences, and satisfaction about online class COVID-19 situations.

### 3.2 Target population and study area

The study intended to represent the problems of online education and the psychological status from the perspective of students’ experience and satisfaction. The primary data has been collected from 60 secondary-level students currently enrolled in different schools in Dhaka city. The study area for this research was secondary level students, particularly from Dhaka city. There are so many schools situated in Dhaka city. Therefore, the findings obtained from the primary data were more suitable to show the student population of Dhaka city. An array of geographic locales was surveyed with school-going adolescents from Mirpur Cantonment and the Dhaka Cantonment area.

### 3.3 Sample collection

There are so many schools in Dhaka city that are using Zoom and Google Meet to teach online. Adolescents who are attending the online class were selected through the process of non-probability purposive sampling. The study concentrated on the secondary level (class 9–10) students of Dhaka city.

### 3.4 Tools of data collection

For this study, the data was gathered from both primary and secondary sources. The tools which were followed for collecting the secondary and primary data are as follows:

#### 3.4.1 Inclusion and exclusion criteria of secondary data

The secondary data was collected from articles, newspapers, research studies, and websites. This paper used secondary survey data to triangulate the interpretative approach for verification and reliability for comparable results in a broad setting. Secondary data was analyzed in terms of theme, pattern, and perspective. The data was compared and contrasted using these themes and patterns, which were linked to relevant literature. The researchers searched published studies and literature of Google Scholar, PubMed, Scopus, and Web of Science for population-based original studies. The initial search was executed using a combination of the following terms: "adolescent," OR "teenager," OR "COVID-19," OR "mental health," OR "school-going," OR "lockdown," "psychology," OR "anxiety" or "depression," OR "Bangladesh." The studies included in the quantitative and qualitative analyses, review articles, and reports highlighting various mental health needs of adolescents and factors affecting psychological health conditions, behaviors, and barriers to mental healthcare were published until December 2021. In order to minimize the incidence of missing articles relevant to this scoping review, the researchers did not filter by year during our search. Conference proceedings, non-English publications, and studies were also eliminated, where the entire paper was unable to be accessed.

#### 3.4.2 In-depth interview

An in-depth interview effectively gathers detailed information on a specific topic beyond the initial and surface-level answers. The collected primary data from the interviews of school-going adolescents was carried out one month into the midst of the COVID-19 surge, considering health protocols and guidelines from the World Health Organization (WHO). The factors behind the impact of COVID-19 on mental health were found from the interviews. The factors were arranged into themes. The study used a set of semi-structured questionnaires for interviewing the school-going adolescents. Two sets of unstructured questionnaires were used separately for conducting in-depth interviews with respondents. The researchers used a mask during the pandemic to conduct the interview and maintained a 5m distance between the interviewer and the interviewee. We have prepared a form of consent. From an ethical point of view, we have first taken permission from the parents as the adolescents are yet to be 18 years old. Those who faced difficulties while responding during interviews found that their parents assisted them. Participants were able to look at the information sheet and ask questions before the interviews. This study's respondents were adequately informed of the things included in the data collection questionnaire before initiating the meeting. The perspectives of the participants were constantly balanced to identify comparisons. As the study progressed, variations and patterns of data emerged, leading to an accurate study that helped to guide the researcher in the direction of the subject or person to be further examined to determine or contrast the progress of the data analysis. Furthermore, the interviews were conducted in the Bangla language based on the preferences of the interviewee. These were transcribed verbatim and then translated into English. Individual coding was done by manually applying to a word processing program.

### 3.5 Ethical consideration

All the interviews have been carried out with informed online written consent, and the respondents were informed. All procedures performed in studies involving human participants followed the ethical standards with the 1964 Helsinki declaration and its later amendments or comparable ethical standards.

## 4 Results

### 4.1 Demography

The respondents were male (30) and female (30) out of 60 respondents. They were in the age group between 13 and 15 years old; all were students from primary and secondary schools. They came from different schools in Dhaka city. The secondary level (class 9–10) students were from Mirpur Cantonment Public School and College, BAF Shaheen College, Dhaka, and BAF Shaheen College, Kurmitola. These schools are conducting their online classes (see Table 1).

**Table 1:** Participants demographic.

Demographics	Frequency (n = 60)
Gender	
Male	30
Female	30
Education (secondary level)	
Class 9	32
Class 10	28
Age	
13–15	60
Attending Online Classes	
Yes	60
No	0

### 4.2 Impact of mental health of school going adolescents

COVID-19 has created a severe impact on every sphere of life. Even adolescents could not escape its devastating impact. From March 18, 2020, all educational institutions are closed. Most school-going adolescents are staying at home without meeting their friends and teachers at school. Even if they are not allowed to play outside with their neighboring friends. And the home-based online system is a whole new thing for adolescents. These might seriously affect adolescents.

**Figure 1:**
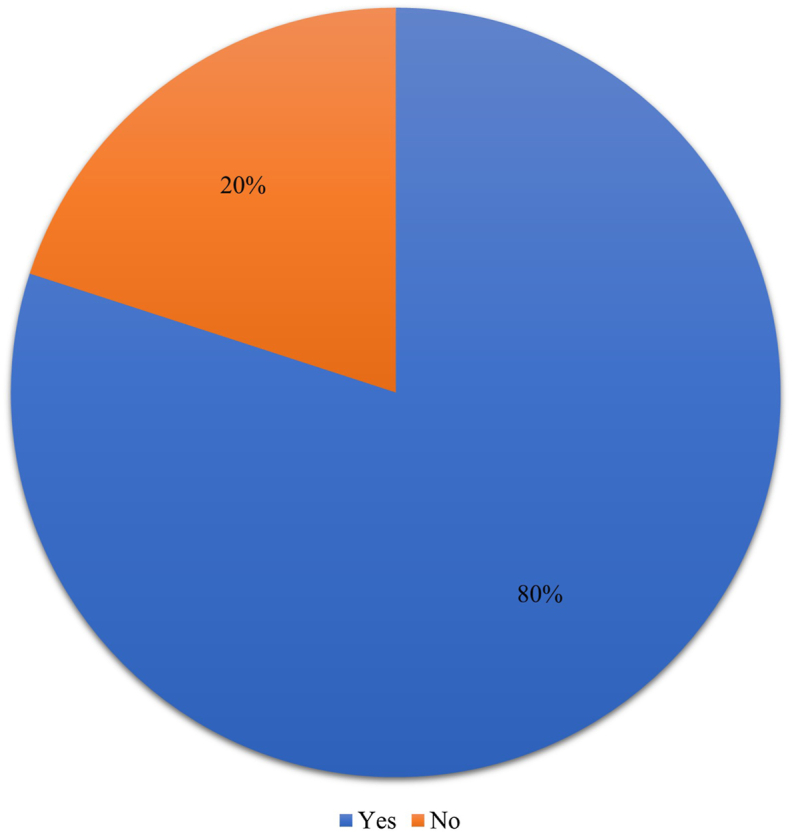
No of adolescents feeling stressed due to COVID-19 (Source: Authors generated using Microsoft Excel).

The study interviewed 60 adolescents, and out of those, 48 adolescents (80%) responded yes to the question if they or their adolescents have felt stressed about the COVID-19 pandemic. Figure 1 shows the number of adolescents who have felt stressed about the COVID-19 pandemic.

**Figure 2:**
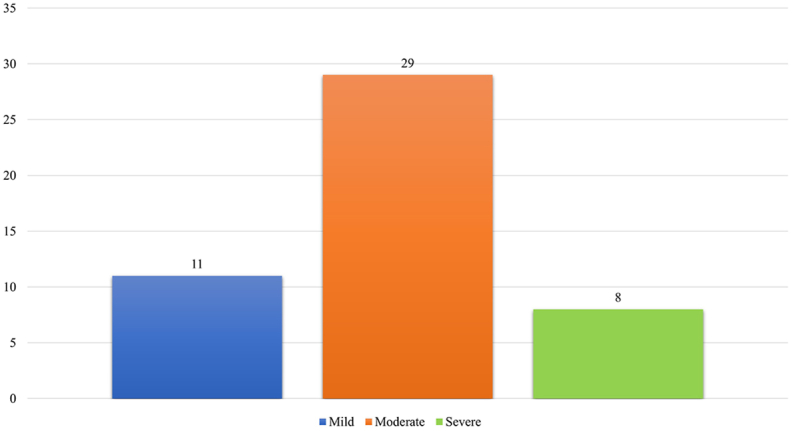
Stress level of adolescents (Source: Authors generated using Microsoft Excel).

Among the adolescents who felt stressed about the COVID-19, 8 adolescents felt severe stress about the COVID-19, 29 adolescents felt moderate stress, and 11 adolescents felt mild stress about the COVID-19. Figure 2, as follows, represents the level of stress of adolescents due to COVID-19.

**Figure 3:**
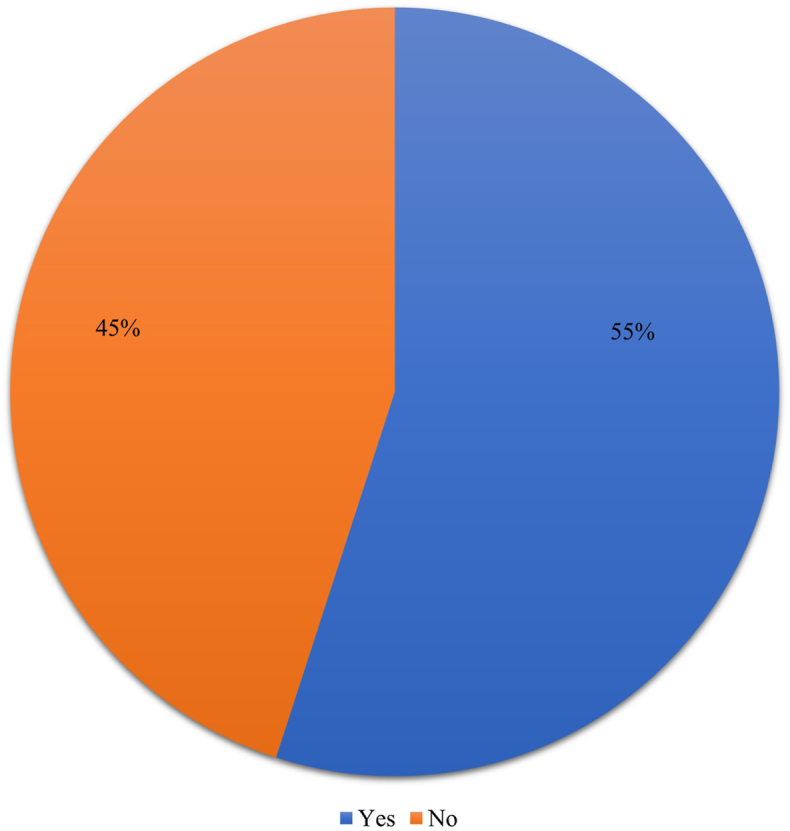
No of adolescents feeling anxiety in lockdown (Source: Authors generated using Microsoft Excel).

**Figure 4:**
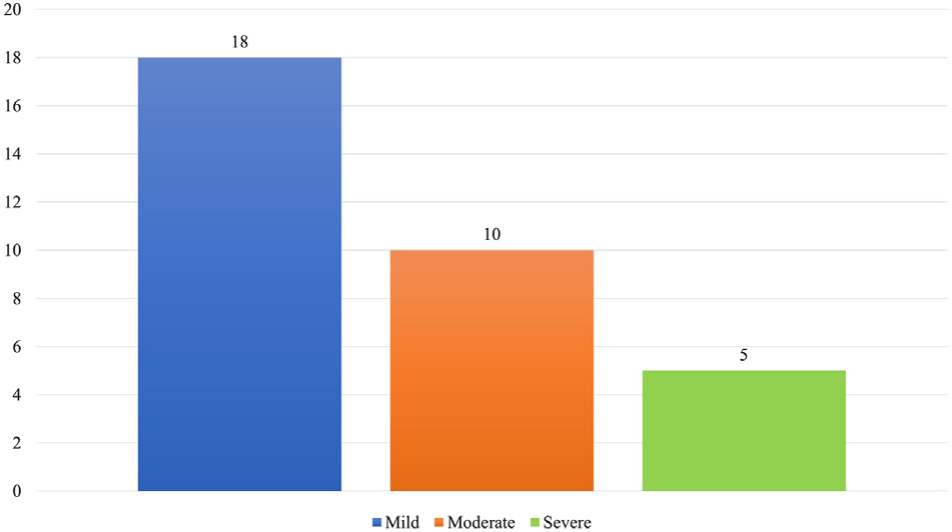
Level of anxiety among adolescents (Source: Authors generated using Microsoft Excel).

The adolescents were asked if they felt anxiety, depression, or had any sleeping disorders during the lockdown. The level of the symptoms was also rated by them. Among 60 adolescents, the number of adolescents who felt each symptom and their level of severity for each symptom is shown as follows:

Figure 3 shows that among the 60 adolescents, 33 adolescents (55%) felt anxiety during the lockdown regarding COVID-19 fear and online classes. The level of adolescents' anxiety is shown in Figure 4.

**Figure 5:**
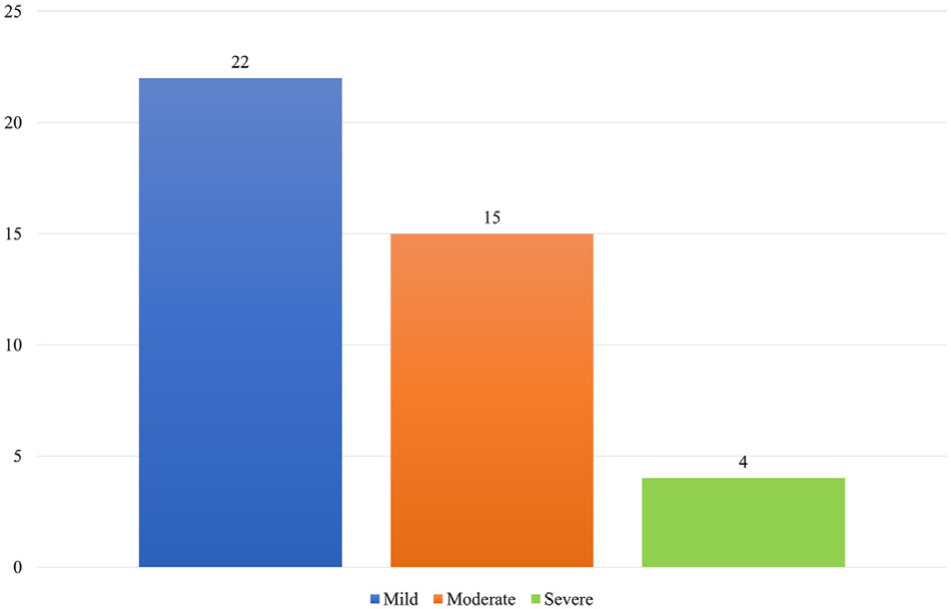
Level of depression among adolescents (Source: Authors generated using Microsoft Excel).

The respondents stated that they felt depressed at a different level of severity. Among 60 adolescents, 41 adolescents felt depressed at a different severity level, and the following Figure 5 shows the number of adolescents who felt depressed during the lockdown.

**Figure 6:**
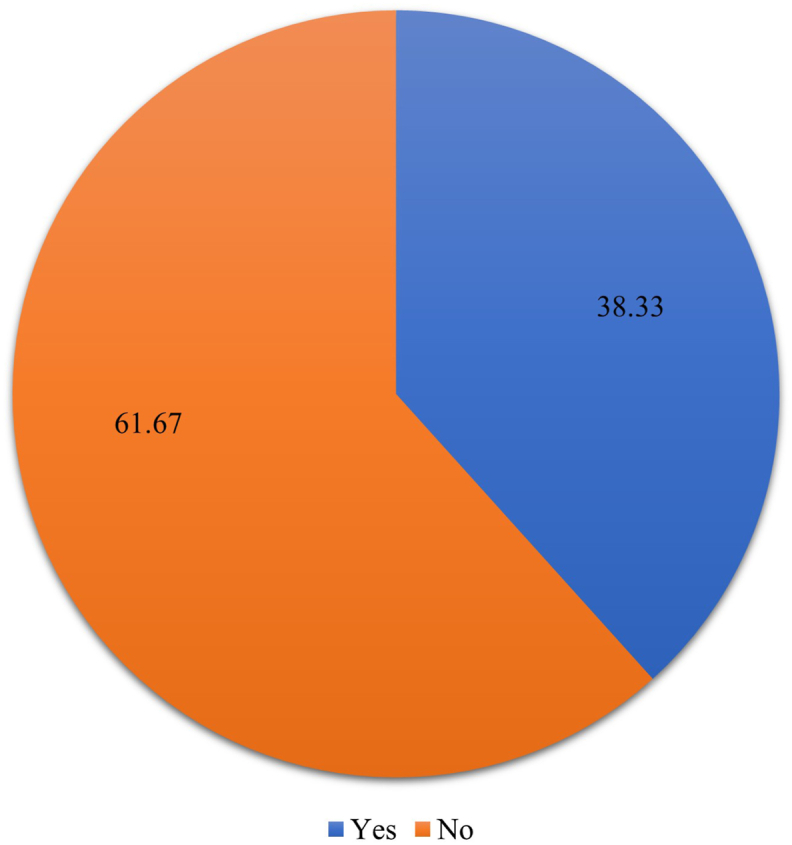
No of adolescents suffering from sleeping disorder (Source: Authors generated using Microsoft Excel).

Moreover, 37 (61.67%) out of 60 adolescents reported having a sleeping disorder during the lockdown. Figure 6 shows the number of adolescents facing sleep disorders.

The following factors have been recognized as impacting the mental health of the population during COVID-19:

#### 4.2.1 Fear of the disease

Young adolescents have never heard of this kind of disease before. The fear of unknown diseases filled their minds with dread. Moreover, there is no widely available vaccine in Bangladesh. On top of that, adolescents will be in the last line to be vaccinated. Therefore, the adolescents felt stressed about this disease if they got infected or even died. They even felt extremely worried about their parents, especially those who had to work and go out on a day-to-day basis. They were anxious about their parents’ death due to COVID-19. Ismat Zahan Oni (pseudonym), aged 13 years old, mentioned:

*“My mother is a nurse at a hospital. She has to go to hospitals every day. I am afraid she might get infected by COVID and get sick. For safety measures, she keeps a distance from me. I want everything to be normal again and hug my mother without any fear.”*

Another secondary school student Fahad Chowdhury (pseudonym)said:

*“My father is a kidney patient. He goes to the bank daily. I really fear if he gets affected by Corona and something happens to him. Whenever he feels sick, even a little bit, I become highly anxious if he has been infected by the virus. This fear gives me continuous mental pressure.”*

Social media and the internet publish different kinds of news. Some are fake, some are true. Adolescents often get confused about which ones to believe and which ones do not. That disturbs their little minds. Many adolescents were not allowed to meet their parents, who were COVID-positive and isolated. Because of this reason, they felt a great deal of stress. It was tough for the adolescents to strictly maintain the safety measures, like washing their hands and wearing masks. Adolescents often complain to parents that they are tired of washing their hands and wearing masks. Adolescents who lost their close ones or someone from their neighborhood felt anxious for themselves and their parents. They might be affected by COVID-19.

10^th^ grade student Rehnuma (pseudonym) said:

*“I cannot go outside for a drink because of the lockdown. I spend time on social media. But the huge amount of ambiguous news on social media makes me more confused about what is accurate information and what is not. As a result, I always remain under stress and get afraid when someone from outside visits us. I am afraid and stressed about the disease.”*

#### 4.2.2 Prolonged closure of school

According to the Global Partnership for Education, if the school provides a balanced education that helps to promote a child's psychological and mental growth (Global Partnership for Education, 2021). School is also important for the socialization of adolescents. It is where they can meet their friends, play with them, learn new things, and do activities. But with the closure of schools, adolescents were deprived of these opportunities. To maintain social distance, the government of Bangladesh has declared to close all kinds of educational institutes from March 18, 2020. This was done to ensure the safety of the general public and adolescents. Adolescents are now spending more than 11 months at home. Many reported that they used to feel very bored and sometimes alone in the initial days of the lockdown. They are upset because they cannot meet their friends and playmates. Even in the initial days, they were not even let go to play in the neighborhood. This really upset their minds. The context is very common for adolescents who live on the rural side or in small towns. However, the urban adolescents felt mostly upset because they could not go to school.

Tanah (pseudonym), aged 14, mentioned that:

*“Sometimes I feel very frustrated about why I cannot go to school and play with my friends. I am imprisoned all the time at home. I feel lonely and bored. I do not like playing video games all the time at home.”*

Most of the respondents mentioned that their daily routine is severely affected by the closure of the school. Their daily eating hours and sleeping hours have changed. Some of the adolescents reported difficulty sleeping. They cannot fall asleep easily as they used to before the closure of the school. The reason they used to be tired and exhausted after all-day school. School even provided them a chance to engage in physical activities. That resulted in sleeping disorders for some.

#### 4.2.3 Disruption in education

Saha et al. (2021) and Bhuiyan et al. (2020) stated that e-learning or distance learning has gained priority in the education sector during the current COVID-19 pandemic situation. Social media such as Facebook, Messenger, and YouTube, Zoom, also gained immense popularity during these quarantine days. These tools are handy in a situation like this, but none of them can provide education as well as the classroom can. Many secondary schools have started online-based learning, though online-based learning was less prominent at the primary school level, as seen from the sample of the research population.

Many adolescents reported they could not provide attention in the online-based class. Moreover, there are various kinds of disruption in the online-based class. Ahmed (pseudonym) studying in class nine said:

*“I feel highly disturbed when there are network falls suddenly in during the class. Often, we can hear what others are saying due to bad network connections. On the other hand, if mobile data finishes or load shedding occurs, students cannot join the class. The same thing happens to teachers.”*

Many of the parents fired home tutors due to vacation or lack of income. From the interviews, most of the students almost forgot what they had learned in the previous class. Adolescents do not want to sit down to study as they have no homework. On the other hand, adolescents reported that parents pressure them to study, and they often feel disturbed. Then again, the date of the Secondary School Certificate (SSC) exams is postponed now and then. Mehreen (pseudonym), an SSC examinee, stated:

*“We are confused about whether there will be an exam or not. This kind of uncertainty makes me anxious as well as demotivated for studying.”*

The evaluation system of online classes often disturbs the psychological health of adolescents. Students feel immense pressure while submitting within a limited time. Adolescents whose parents have less technical knowledge face high difficulty in continuing their online education. The environment at home is not often suitable for learning. Many houses do not have separate rooms for adolescents. The presence of other family members and young adolescents often creates an embarrassing situation for them. Raihan (pseudonym) was mentioned:

*“I feel ashamed when my sister comes before the camera when the classes are going on. All the students laugh at me. I wish I had a separate room.”*

Another SSC examinee, Badhon Rahman (pseudonym), stated that:

*“I have my eyes problem, so I cannot give proper attention to my online classes. I worry about my results.”*

A student named Rita (pseudonym) aged 14 reported that:

*“I felt upset because I cannot cope up with online classes. Most of the time, I did not get what my teacher was talking about. So, I could not do well in my exams. It makes me feel worried.”*

#### 4.2.4 Digital divide among adolescents

The term "digital divide" was introduced in the mid-1990s and defined as the gap separating those who have access to new forms of information technology from those who do not (Srinuan and Bohlin, 2011). It can be said that COVID-19 has created a form of digital divide among adolescents. Among the schools' samples in urban or small-town areas, almost all have started online-based classes or assignment-based evaluation systems. Some adolescents are deprived of this kind of advantage. A student named Shimu (pseudonym) said:

*“I do not have any personal devices for online classes. In my family, only my father has a laptop for his office tasks. My mother's phone does not support long-hour use of the phone. My parents are trying to afford me a new device only for online classes and tasks. I am suddenly feeling disconnected from the other students. Due to a lack of device, I cannot engage in online classes regularly.”*

Neither teachers nor students have enough technical skills or resources to practice online-based learning in schools like this. Some students do not even have access to digital devices. Many students reported that they had not even heard about online classes. They have already forgotten what they have learned so far. Therefore, some students are falling behind. Moreover, it might create a digital divide between the students within the same schools. Some students from the schools find it frustrating that they have not accessed digital devices. It creates a significant problem for the mental health of adolescents. Labiba (pseudonym) said that:

*“My friend can attend the online classes. But there is no notice of classes in our school. I feel upset sometimes. I am lagging behind”.*

#### 4.2.5 Loss of income of caregiver

According to a Business Insider (2020) report, in 2019, poverty elasticity showed that 20% of people were under poverty. The poverty rate will jump from 20% to 40% due to this COVID-19 pandemic. More than 9% of people will be in food poverty. The vulnerability index will jump from 20% to 35%. The socioeconomic determinants of a crisis can affect adolescents' development and mental health, and their effects may even persist into adult life, regardless of whether financial circumstances improve (Saha et al., 2021). Most of the respondents mentioned that their parents lost their income source. There was reported chaos in the family, which mentally disturbed the adolescents. A few students' families earn income from their brothers' or sisters' private tuition. They lost their tuition, leaving them feeling like a burden to the family. In the words of Jarif (pseudonym) mentioned:

*“My father has lost his job. My brother used to earn money by tutoring. But he lost tuition owing to the pandemic situation. We do not have any income source now, and I am afraid of how we are going to survive.”*

#### 4.2.6 Addiction to digital device, video games, and social media

As the schools are closed, students mostly pass their time using digital devices like Android phones and personal computers. They mostly play video games or use social media. They are over-engaged with this device, and on the other hand, fake news on social media is confusing. Most of them mentioned that adolescents are engaged most of the time on mobile phones. They do not stop using their mobile phones even after the online classes. They spent more time on their phones than with their family, siblings, and playing outside. Even if they are not allowed to use mobile phones, they seem disturbed or agitated. Excessive use of social media keeps them away from creating bonds with the family. Family is important for emotional support. Due to their addiction to mobile phones, they are alone, and their socialization process is disrupted. A student named Marium Akter (pseudonym) reported that:

*“During the COVID-19 situation, I was always busy with my mobile phone and playing games. Also, I cannot prohibit them as I cannot go out to play. But access to and use of devices may cause eye problems as well as mental diseases.”*

Another respondent named Sohail (pseudonym) stated that:

*“I keep busy with playing video games. I spent more time on phones rather than spending time with family, siblings, and playing outside.”*

#### 4.2.7 Culture of ignoring mental health of adolescents

Adolescents are less vocal about their mental health problems. It is an irony that adolescents' feelings of anxiety and stress often go unnoticed. They did not have enough mental support systems. Instead, they were often rebuked when they acted out of irritation. No mental health professional was approached. Only in a few instances did parents make their adolescents understand and be well educated about the crisis. Adolescents reported that their parents were angry at them because of their excessive use of mobile phones and gadgets. But using these was just a means to remove boredom and connect with friends. Afsan Pasha (pseudonym) stated:

*“My parents are always angry with me because I use the phone. But I have no other thing to do other than play games on my phone.”*

## 5 Discussion

**Figure 7:**
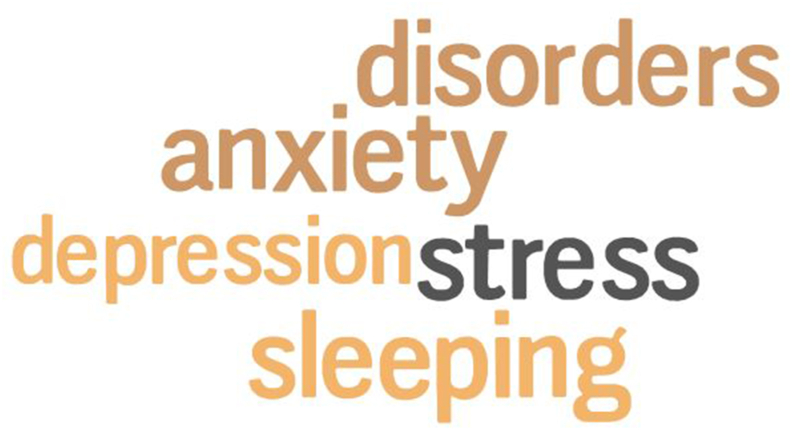
Symptoms of mental health issues among adolescent students during the pandemic (Source: Authors generated by using Microsoft PowerPoint Pro Word Cloud).

Based on the above findings from respondents' responses and in-depth interviews, mental health is essential, but adolescents are less vocal about their mental wellbeing. The major findings reflected the mental health situation of the adolescents, whose mental health needs are often neglected. The study found different types of symptoms like stress, anxiety, sleeping disorders, and depression were faced by the study population at varying levels of severity (see Figure 7).

**Figure 8:**
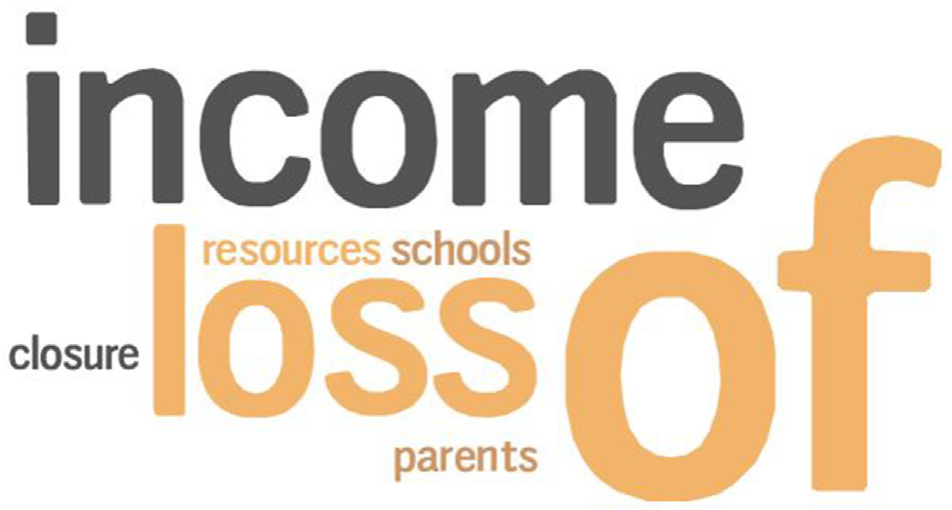
Determinants which affect adolescents' mental health (Source: Authors generated by using Microsoft PowerPoint Pro Word Cloud).

The finding reveals an array of factors that affect an adolescent's mental health. The respondents spoke about the problems and changes in life that impacted their overall mental health. Closure of schools, loss of income of their parents, loss of income resources affected their mental health at different levels of severity (see Figure 8). Many adolescents reported sleeping disorders and depression, but there are no policies to address adolescents' mental health problems in a crisis moment like this.

The influence of the COVID-19 pandemic on four key mental health concerns affecting the general Bangladeshi population and discovered an increase in the prevalence rates of loneliness, depression, anxiety, and sleep disturbance. A greater number of people are mentally ill, with varying degrees of severity (Das et al., 2021; Mallik and Radwan, 2021). During the pandemic in Bangladesh, three fourths of the adolescents were distressed by household stress (Baird et al., 2020). Adolescents are concerned about their studies and exams. Depression is frequently caused by physical and psychological stress, anxiety, frustration, negative experiences, and fear. Because schools are using virtual methods in which tears are required to engage with adolescents online, detecting any behavioral changes can be difficult (Gautam et al., 2022). As a result, parents may find themselves playing the additional role of teacher or counselor, particularly for adolescents who are showing signs of depression. However, they are maybe unaware of such sudden shifts in their adolescent's behavior, which frequently leads to mental disturbance and suicide risk (Erbacher, 2020).

According to Aresfin and Shafiullah (2020), in Bangladesh, mental health has historically been given little importance for adolescents due to the demographic group generally being less vocal about their concerns. The study found a similar kind of response to this fact. Parents and caregivers knew of the symptoms but took no measures to solve this. Even adolescents were reprimanded and even hit by their parents while they were showing irritating behavior. None of the respondents reported seeking professional help despite feeling these psychological symptoms like anxiety, depression, and sleep disorders. That scenario goes with the picture of addressing the mental health culture in Bangladesh.

Moreover, home-based online classes are creating a digital divide. Students who do not have access to digital gadgets or have access but face difficulties, feel immense mental pressure. Then, the submission deadline disturbs the mental health of adolescents. Adolescents are frequently affected by the sudden loss of network signals, the cost of data, and the availability of broadband. The online-based education system should be appropriately revised to give adequate attention to adolescents’ mental health.

Bangladesh's social safety net programs cover some aspects of adolescents' wellbeing (Sifat, 2021b; Sifat et al., 2022). But the mental health of adolescents is not well reviewed. Adolescents' mental health should be given the utmost attention like any other problem covered by social safety nets. It is found in the literature that socio-economic determinates of any crisis create problems for the mental health of the population, especially in adolescents. The COVID-19 pandemic is a crisis, and one of the socio-economic determinants of the pandemic was the loss of income of people. The loss of income impacts overall well-being, including adolescents' mental health. The study also corresponds with this statement. The study found that adolescents whose parents lost income had to deal with chaos in the family or did not have enough family support.

### 5.1 Recommendations

#### 5.1.1 Strategy to protect adolescent mental health during pandemic

Due to the COVID-19 pandemic, fear, anxiety, sleep disturbances, depression, and suicidal thoughts spread among Bangladesh's population (Ahmed and Sifat, 2021a; Sifat et al., 2022). It is high time for the government to take the necessary steps to address mental health issues, such as arranging for e-therapy or online counseling. To assist individuals suffering from mental distress, the government, non-governmental organizations, charitable organizations, and youth-led enterprises should provide free tele-counseling and video-counseling. Along with online classes, online counseling should be organized. Counseling for both parents and students is essential. It is also essential to urge teachers to monitor students during online classes. Authorities should create a clear set of online class and evaluation guidelines to reduce uncertainty and anxiety. In addition, educational institutions could appoint psychologists to help mentally unstable students. In the long run, the government can provide funding to educational institutions in order to construct counseling centers. Furthermore, the government may support mental health programs for students. Communicating with family and friends will be a primary means of coping with stress and anxiety during the COVID-19 crisis. Another recommendation is to regularly utilize a virtual gathering program like Zoom or Google Meet to communicate with friends and family. In partnership with the government, the school administration should establish a mobile mental health service application to allow students to receive therapy from a trained psychiatrist or psychologist.

More resources should be allocated to the educational institution to prepare for increased student requests for mental health services. We encourage schools to provide academic program-based interventions and services to enhance institutional mental health strategies. We encourage administrators to develop innovative ways of improving students' mental health and reducing stress. Many non-governmental organizations have committed themselves to mental health advocacy and awareness, and we encourage administrators to work with them to develop interventions, proactive programs, and extend existing services. There is a need for widespread educational initiatives to improve people's perceptions of mental health and prevent discrimination against those with psychological conditions.

##### 5.1.1.1 Role of caregivers

The role of the parents is the most crucial factor in avoiding the detrimental effect of COVID-19 on their lives. Parents have to spend as much time as possible with their adolescents. They should help them understand what COVID-19 is, try to alleviate their fear, and make them aware instead. Parents should know that their adolescents are going through a different phase that they have never seen before and should understand their perspective. If their children are too negative, they should not hit them. If needed, they should seek the help of professionals. From time to time, they can meet their cousins or nearby friends with the best safety measures as much as possible. They should introduce the adolescents to better online games or apps that are educational. Some other points to be noted:

First, exposure to fake news and continuous media news about COVID-19 should be limited. It hampers their mental health. Limiting these might help to reduce the fear of infection.

Second, indoor game activities can be increased more so that the adolescents have a good time. It will relieve them of the anxiety.

Third, efforts should be made to maintain a continuous routine for adolescents with a fixed bedtime. Parents should try to ensure adolescents are getting involved in physical activities.

Fourth, exercise and yoga are good for overall health. Parents should be role models for adolescents and try to include them in their daily lives and their adolescents’.

Fifth, creative activities like music, art, crafts, etc., will calm their minds and help them divert from the dread of a pandemic and boredom.

##### 5.1.1.2 Role of community and governments

First, online mental health services like tele-counseling and video counseling, which provide mental health services to adolescents over the phone, should be extended. This type of program should be done on a community level, at least on a district level.

Second, a psychological counselor should be appointed in every school; this can only happen if investment in mental health increases as suggested above.

Third, the national education syllabus can include a chapter where adolescents can learn about mental health, especially in crises like the COVID-19 pandemic.

Fourth, national television programs can broadcast shows consisting of experts in child psychology and psychiatry about addressing adolescents' mental health in COVID-19. It will create awareness among adolescents and parents. Also, these types of programs should be incorporated into the national programs in times of crisis.

Fifth, a yearly seminar can be arranged to educate the teachers and parents on how to take care of their adolescents in a crisis like this.

Sixth, schoolteachers should be instructed to talk about problems and crisis situations to prepare the students mentally for such a situation as much as possible.

Seventh, the teacher can communicate and ask about the lives of adolescents, make them aware of this deadly disease, and try to reduce the confusion.

## 6 Limitation of the study

This analysis raised several issues that must be considered while assessing the data. First, the sample size was relatively small. The interview was carried out one month into the midst of the COVID-19 surge, considering health protocols and guidelines from the World Health Organization (WHO). Second, we employed non-probability purposive sampling to recruit respondents, which hinders the capacity to generalize the results to the larger population. On the other hand, there was insufficient literature available in the country's context on this topic. Therefore, literature on the same field but in another country's context was attempted. There was an issue with tight scheduling during the study because of strict lockdown. Time constraints are one of the major limitations of the study. Rather than that, in many cases, in-person interviews were not possible due to the COVID-19 pandemic, and there was an availability problem with large respondents.

## 7 Conclusion

Lockdown as a means to stop the spread of COVID-19 has posed a threat to adolescents' mental health. This unexpected lockdown has caused enormous disruption to daily routines for the global community, especially adolescents. Most schools closed, canceled classes, and moved their students to home-based or online learning. Lockdown has created day-to-day problems, including playing with friends, going to school, meeting friends, sharing with friends, disruption and uncertainty in education, loss of income for parents, etc., which has had a detrimental effect on adolescents' mental health. The guardians, educational institutions, and health authorities must consistently protect adolescents' mental health through open communication and facilitate professional counseling to address stressors. Additional attention should be given to adolescents who are more vulnerable to the mental health crisis through a collaborative approach involving their parents, educators, school administrators, counselors, psychologists, and psychiatrists. The lesson from this study is to get more mindful of the effectiveness, perception, and difficulties of school-going adolescents in order to prevent students from developing severe mental health issues. As mental pressure may lead to various illnesses, health experts should recommend implementing tele-counseling services during this pandemic. These included redressing systemic underinvestment in psychiatric care; delivering "emergency mental health" through online support, such as tele-counseling for frontline health staff; and engaging proactively with people reported to have depression, anxiety, and mental stress problems. But it is high time to give the problem its required highlight to handle the current unforeseen situation. Further studies are encouraged to investigate the adolescents' psychological impacts of COVID-19 in all other cities in Bangladesh.

## Declarations

### Author contribution statement

Ridwan Islam Sifat: Conceived and designed the experiments; Performed the experiments; Analyzed and interpreted the data; Contributed reagents, materials, analysis tools or data; Wrote the paper.

Maisaa Mehzabin Ruponty: Conceived and designed the experiments; Performed the experiments; Contributed reagents, materials.

Md. Kawser Rahim Shuvo; Shidratul Moontaha Suha: Conceived and designed the experiments; Contributed reagents, materials, analysis tools or data.

Mehjabin Chowdhury: Conceived and designed the experiments; Performed the experiments; Contributed reagents, materials, analysis tools or data; Wrote the paper.

### Funding statement

This research did not receive any specific grant from funding agencies in the public, commercial, or not-for-profit sectors.

### Data availability statement

No data was used for the research described in the article.

### Declaration of interests statement

The authors declare no conflict of interest.

### Additional information

No additional information is available for this paper.

## Appendix A Supplementary data

Supplementary material_Questionnaire.docx Supplementary material_Questionnaire.docx
