# Supplementary material for: Impact of COVID-19 pandemic on the mental health of school-going adolescents: insights from Dhaka city, Bangladesh
Source: Heliyon. 2022 Mar 29;8(4):e09223. doi: 10.1016/j.heliyon.2022.e09223 (PMC8963972; doi:10.1016/j.heliyon.2022.e09223)
Supplement: Supplementary material_Questionnaire.docx [file mmc1.docx]

**APPENDIX: Questionnaire**

Demographic Information of the Respondents:

1. Age:
2. Gender: Male Female
3. Education level:  Class 9  Class 10
4. Do you attend online class?  Yes  No
5. What is your guardian’s occupation?

teacher farmer businessmen  government officials informal job rickshaw-puller  homemaker  health worker  others…….

Basic information on Study Topic:

1. Do you ever feel stress about COVID-19?

Yes  No

1. Do you ever feel stress interferes with studying, daily living activities or social activities?  no problem with mental stress mild problems  stress causes me moderate difficulty severe stress
2. Do you ever feel anxiety during lockdown?

Yes No

1. Do you have problems with your behavior or fear of anxiety that interfere with your studies, daily living activities or social activities?

No pain mild moderate severe

***In-depth interview questionnaire:***

Set 1:

Q1) Do you notice any changes in your lifestyle due to the COVID-19 situation? If yes, could you please describe those changes?

Probe Questions:

1. Is your sleeping hour same as before or changed?
2. Do you communicate with yours like earlier or how you practicing your friendship relation?
3. How it is with your parents and siblings?

Q2) Do you think COVID-19 has had a negative impact on your life?

Probe Questions:

1. Do you face any challenging situations due to COVID-19?
2. How was it during lockdown?
3. Do you fear COVID-19 and its diseases?
4. Do these situations affect your mind? If yes, can you please describe it?
5. Do you have to attend online classes? If yes, do you face any difficulties attending online classes?

Q3) Do you think COVID-19 has impacted your mental wellbeing? If yes, can you please describe it?

Probe Questions:

1. Do you ever feel stressed?

2.Are you anxious of anything. e.g., online exam attendance/ parents overlook etc?

3. Do you face sleeping disorders or any kind of depression?

Set 2:

Q1) Due to school closure, do you face any kind of challenges in studying and not meeting with others?

Q2) Due to the COVID-19 pandemic, what kind of difficulties are faced by your family? Have those difficulties impacted your education?

Probe Questions:

1. Do financial problems in your family hampered your studies?
2. Do you have enough access to online class materials (network, applications, software, mobile phone/personal computer, or laptop)?

Q3) How do you spend your leisure time?

Probe Questions:

1. Do you use social media applications?
2. Do you play video games during the COVID-19 situation?

Q4) During the pandemic, what do you do to overcome your psychological stress? Do your parents support your mental health issues?
